# Asthma: epidemiology of disease control in Latin America – short review

**DOI:** 10.1186/s40733-017-0032-3

**Published:** 2017-05-11

**Authors:** Dirceu Solé, Carolina Sanchez Aranda, Gustavo Falbo Wandalsen

**Affiliations:** Division of Allergy, Clinical Immunology and Rheumatology, Department of Pediatrics, Escola Paulista de Medicina, Federal University of São Paulo, Rua dos Otonis, 725 – Vila Clementino, 04025-002 São Paulo, Brazil

**Keywords:** Asthma, Asthma contro l, Prevalence, Infants, Children, Adolescents, Latin America

## Abstract

Asthma is reported as one of the most common chronic diseases in childhood, impairing the quality of life of patients and their families and incurring high costs to the healthcare system and society. Despite the development of new drugs and the availability of international treatment guidelines, asthma is still poorly controlled, especially in Latin America. Original and review articles on asthma control or epidemiology with high levels of evidence have been selected for analysis among those published in PubMed referenced journals during the last 20 years, using the following keywords: “asthma control” combined with “Latin America”, ” epidemiology”, “prevalence”, “burden”, “mortality”, “treatment and unmet needs”, “children”, “adolescents”, and “infants”. There was a high prevalence and severity of asthma during the period analyzed, especially in children and adolescents. Wheezing in infants was a significant reason for seeking medical care in Latin American health centers. Moreover, the frequent use of quick-relief bronchodilators and oral corticosteroids by these patients indicates the lack of a policy for providing better care for asthmatic patients, as well as poor asthma control. Among adults, studies document poor treatment and control of the disease, as revealed by low adherence to routine anti-inflammatory medications and high rates of emergency care visits and hospitalization. In conclusion, although rare, studies on asthma control in Latin America repeatedly show that patients are inadequately controlled and frequently overestimate their degree of asthma control according to the criteria used by international asthma treatment guidelines. Additional education for doctors and patients is essential for adequate control of this illness, and therefore also for reduction of the individual and social burden of asthma.

## Background

Asthma is a public health problem worldwide, not just in high-income countries. This illness occurs in all countries regardless of their level of development, being generally under-diagnosed and undertreated, and most asthma-related deaths occur in low-income and lower-middle-income countries [1]. Asthma is reported as one of the most common chronic diseases in childhood, impairing the quality of life of patients and their families and incurring high costs to the health care system and society [2]. Epidemiologic studies have shown that the prevalence of asthma is increasing worldwide, especially in non- industrialized countries, and it is currently estimated that approximately 235 million people suffer from asthma [1].

The current level of asthma control in Latin American countries falls far short of the goals set forth by current international guidelines [3]. Latin America (LA) comprises 20 different countries and is inhabited by 600 million people, with approximately 40 million asthmatics [4]. Latin America (LA) shows great geographic, political and economic inequalities. Except for Brazil, all Latin American regions have Spanish roots. The prevalence and severity of asthma in LA is elevated. Despite these indicators, few countries in the region have healthcare systems able to provide the most adequate treatment for these patients [5–8]. This leads these patients to not having their illness adequately controlled, with high rates of acute exacerbation, as revealed by high frequent searching for urgent care in emergency departments, high hospitalization rates and even a greater number of deaths. It should also not be forgotten that infants with recurrent wheezing also represent a significant portion of asthma patients, corresponding to important expenses. The present study reviews the main available data on asthma epidemiology in LA, especially related to asthma control.

### Epidemiology of asthma

Asthma begins early in life, and existing data on asthma prevalence in LA focuses predominantly on children and adolescents [9, 10]. The International Study of Asthma and Allergies in Childhood (ISAAC) has shown that the prevalence of asthma symptoms varies widely among Latin American countries [9]. The data from ISAAC’s phase 3 (2002/03) indicate that the prevalence of current wheeze in children (6 to 7 years old) ranged from 8.4% in Mexico to 37.6% in Costa Rica [9]. Among adolescents (13 to 14 years old), the prevalence of current wheeze ranged from 11.6% in Mexico to 30.8% in El Salvador (Table 1) [11]. Another important observation from ISAAC phase 3 was the confirmation that asthma in LA countries is under-diagnosed, since the prevalence of physician-diagnosed asthma, ranging from 6.9% (Mexico) to 33.1% (Peru), was lower than the prevalence of current asthma in 10 of the 17 LA countries participating in the study (Table 1) [11].

**Table 1:** Prevalence of symptoms of asthma obtained by the International Study of Asthma and Allergy (ISAAC) Phase 3 in adolescents from different countries of Latin America [11]

Country	Centers	N	Current asthma (%)	Asthma ever (%)
Argentina	4	12,716	12.5	9.3
Bolivia	1	3257	13.5	12.3
Brazil	20	58,418	18.7	13.3
Chile	5	13,793	15.3	15.1
Colombia	3	10,134	11.8	14.2
Costa Rica	1	2436	27.3	23.2
Cuba	1	3026	17.8	30.9
Ecuador	2	6096	16.6	10.9
El Salvador	1	3260	30.8	24.0
Honduras	1	2675	22.0	18.3
Mexico	10	29,723	8.7	6.9
Nicaragua	1	3263	13.8	15.2
Panama	1	3183	22.9	20.5
Paraguay	1	3000	20.9	12.8
Peru	1	3022	19.6	33.1
Uruguay	2	4915	16.4	17.0
Venezuela	1	3000	15.4	29.7
Region total	56	165,917	15.9	13.6

Data for asthma in adults in LA are rare. A national study in Colombia [12] indicated a prevalence of 6.3% of adults with a medical diagnosis of asthma, while a study in Mexico [13] found a prevalence of 5%.

Data for asthma morbidity and mortality in LA are also insufficient and limited to isolated population groups. Asthma mortality is assumed to be high in LA. A pioneering study assessed asthma mortality (for ages 5-34) in Argentina, Brazil, Chile, Colombia, Costa Rica, Cuba, Ecuador, Mexico, Paraguay, Peru, Uruguay, and Venezuela between 1990 and 1997. This study identified important differences in the average mortality rates observed during this period for different countries, varying between 0.28 deaths/100,000 inhabitants (Chile) and 1.35 deaths/100,000 inhabitants (Costa Rica), with higher predominance among women and in areas located in more southern parts of the continent [14]. This study found that many deaths from asthma, particularly in Chile, Argentina and Paraguay occurred at home and not in a hospital environment, indicating that these patients may suffer from a lack of access to medical care or medical monitoring [14].

In Brazil, a temporal assessment of mortality rates from asthma in patients aged 5 to 34 years-old, between 1980 and 2010, documented a significant increase in mortality rates during the first 12 years, rising from 0.55 deaths/100,000 inhabitants in 1980 to 0.65 deaths/100,000 inhabitants in 1997, followed by a period of stability and then a tendency of decreasing rates between 2000 and 2010, reaching a rate of 0.44 deaths/100,000 inhabitants [15]. Differences in mortality rates were observed according to age groups and higher values were noted for states in the southern region of the country [15]. The same pattern of decline in asthma mortality was observed in another study in children under 5 years of age, starting with 5 deaths/100,000 inhabitants in 1980 and reaching 0.85 deaths/100,000 inhabitants in 2007 [16]. This reduction may possibly have occurred due to an improvement in the knowledge of asthma management by doctors and patients, as well as due to the implementation of a government asthma program, which provides free inhaled corticosteroids for patients with severe asthma. These data are reinforced by what has been observed in Salvador (Bahia, Brazil) after implementation of the Program for Control of Asthma and Allergic Rhinitis (ProAR - *Programa para o controle de Asma e Rinite Alérgica*), which prioritized the control of severe asthma. This program brought a reduction in asthma-related costs to the public health system (US$ 387 per patient/year) and to families (US$ 789 per patient/year) [17]. However, asthma is still one of the twenty most common reasons for primary care visits in Brazil, representing the third leading cause of hospitalization within the Brazilian public health system (SUS - *Sistema Único de Saúde*) [18, 19].

A reduction in rates of mortality from asthma has also been observed in Costa Rica after the creation of the National Asthma Program (NAP), which provided inhaled beclomethasone for all asthmatic patients [7].

### Wheezing in the first years of life

Wheezing during the first years of life is a highly important topic due to its frequency and the financial and emotional burden it places on families and health systems. Asthma begins most often during this period of life, being under-diagnosed and consequently inappropriately treated and therefore compromising the quality of life of patients and their families. According to the Global Initiative for Asthma (GINA), recurrent wheezing (3 or more episodes) is being used as a diagnostic criterion (i.e., synonym) of asthma in infants and preschool children [2]. A pioneering study, the International Study of Wheezing in Infants (EISL - *Estudio Internacional de Sibliancia en el Lactante*) [20] made it possible for the first time (EISL-1) to identify the prevalence and factors associated with wheezing in infants living in European and Latin American centers during their first year of life [10]. The comparative analysis between affluent (Europe) and non-affluent countries (LA) documented a higher prevalence of recurrent wheezing and more severe episodes (i.e., higher rates of visits to urgent care centers and hospitalizations due to wheezing and higher consumption of medications) among centers in LA [21]. These data confirmed previous observations in small population groups in Chile, Honduras and El Salvador, where the highest frequencies of recurrent wheezing were found in infants with lower socioeconomic status [22, 23].

The most recent version of the International Study of Wheezing in Infants (EISL-3), carried out seven years after the first study, included interviews with the parents of 12,405 infants from 11 centers in six South American countries (Argentina, Brazil, Chile, Colombia, Peru and Uruguay), and found a high average prevalence of recurrent wheezing (16.6%), with wide variations among the participating centers (7.5% in Bucamaranga, Colombia and 26.7% in Montevideo, Uruguay) [24]. The comparative analyses between centers which took part in EISL-1 and EISL-3 (São Paulo and Curitiba, Brazil; and Santiago, Chile) showed a maintenance of previously observed levels (~22.0%), although there was an increase in severity levels in EISL-3, which, similarly to EISL-1, indicated a high economic burden on parents and public health systems, frequent use of emergency care centers, high rates of hospitalization (due to severe wheezing episodes or pneumonia) and overuse of medications, particularly oral corticosteroids and antibiotics [10, 24].

Despite difficulties inherent to this age group, many of the infants had been previously diagnosed as asthmatic by a doctor during their first year of life, as already seen in EISL-1. The average prevalence of medical diagnosis of asthma in EISL-3 was 4.5% (with prevalence ranging from 2.6% in Cuiabá, Brazil to 9.7% in São Paulo, Brazil) [24], and children with a medical diagnosis were those with most severe wheezing. Nonetheless, the individual analysis for each center revealed that all patients with a medical diagnosis of asthma in São Paulo had been treated with short-acting beta 2 agonists and oral corticosteroids, while 22.0% of these patients received inhaled corticosteroids as a form of anti-inflammatory treatment, revealing the inadequate treatment that these patients were receiving [25]. Moreover, the inadequate control of these patients may explain the higher of complications found among them, such as high rate of hospital admissions due to wheezing, medical diagnosis of pneumonia and even hospitalizations due to pneumonia [24, 26].

Based on the above, we may conclude that lower socioeconomic status may be associated with more severe forms of wheezing. This may be a result of impaired access to the health system by these patients, lower frequency of medical care and consequently less opportunities for long-term anti-inflammatory treatment, which makes these patients more prone to acute exacerbations, more frequent visits to urgent care centers, higher hospitalization rates and higher consumption of oral corticosteroids, incurring higher costs for the country and the health system. In general terms, we can infer that the control of wheezing during the first year of life is a significant marker of the level of control of respiratory diseases in a given locality.

### Asthma control

Despite advances in asthma treatment and in the implementation of guidelines for asthma management, the disease remains poorly controlled, especially in non-affluent countries [2, 4, 27]. Lack of patients’ access to health care, lack of asthma diagnosis, inappropriate treatment and patients’ failure to take prescribed medications properly even after receiving instructions, whether because of a lack of understanding or a lack of adherence to treatment, are some explanations for this failure [2, 28]. Adherence to treatment is fundamental for asthma control, and many factors can interfere with it: knowledge about the disease, cultural standards, socioeconomic factors, lack of perception of asthma symptoms, adverse events, and ability to use medicaments (inhalers) may all represent factors interfering with asthma control [2, 29].

Although the GINA has recommended an ideal treatment and monitoring protocol for asthma to be applied worldwide, local limitations have made it unfeasible to be implemented without restrictions. The GINA indicates as long-term goals for asthma a good control of symptoms, as well as minimization of future risks of exacerbations, fixed airflow limitation and adverse effects of treatment [2]. It should be mentioned that what is recommended in treatment guides often cannot be put into practice in real life. With this in mind, many studies have been performed in order to better understand the impact of these guidelines for asthma management.

Studies assessing asthma control in Latin American patients are rare and mostly limited to the adult population. The Asthma Insights and Reality in Latin America (AIRLA) survey [30] was the first to analyze, between May and July 2003, the quality of treatment and asthma control in LA, assessing health care utilization, the severity of symptoms, activity limitations, and medication use. From a total of 46,275 families interviewed on the telephone, 2184 asthmatic adults or parents of asthmatic children (under 16 years old) from 11 LA countries (Argentina, Brazil, Chile, Colombia, Costa Rica, Ecuador, Mexico, Paraguay, Peru, Uruguay and Venezuela) were selected to take part in the study according to established standards [31, 32]. The results obtained were worrisome, showing that asthma control in LA falls short of goals defined in international guidelines, since 56% of the respondents had daily symptoms, 51% experienced nocturnal awakenings, and over half of all respondents had been admitted to hospital, sought urgent care or unscheduled visits to healthcare facilities due to asthma during the previous year. 79% of adults and 68% of children reported that asthma symptoms limited their activities, and absence from school and work due to asthma was reported by 58% of children and 31% of adults, respectively [30].

Moreover, self-reported perception of asthma control did not match symptom severity, even in patients with persistent severe asthma. Although 37% of the patients reported treatment with prescription medications, only 6% were using inhaled corticosteroids. Even though 44.7% of the patients reported having their illness well- or totally-controlled, only 2.4% (2.3% of adults and 2.6% of children) reached all criteria of total asthma control according to the GINA [33].

As an extension of this study, the authors used the same database to assess the cost of unscheduled visits to healthcare facilities according to the different degrees of asthma severity. Search for unscheduled visits was significantly more frequent among patients with severe, persistent symptoms (71.9%), but was not negligible among patients with mild, intermittent symptoms (45.7%). From an economic point of view, these unscheduled visits represented 73.2% of the expenses on asthma-related care in the participating countries. These expenses were higher among adults (over 16 years old) and children (under 16 years old) with persistent severe asthma (US$ 558 and US$ 769, respectively) in comparison to patients with persistent mild or intermittent symptoms (US$ 204 and US$ 215, respectively). This follow-up study, therefore, confirmed the high costs incurred by uncontrolled asthma on health resources in LA [34].

The need to obtain more complete and comprehensive information, such as general patient expectations and unmet needs regarding the current state of their asthma treatment, as well as the need for a standardized instrument which might be applied in different parts of the world led to the creation of the Asthma Insight and Management (AIM) survey [35].

The AIM study was designed to explore and document asthma-related patient perceptions, behaviors, and presentation patterns, as well as recent trends in asthma management [36–38]. The survey consisted of 53 questions covering five asthma-related topics: 1) symptoms; 2) impact of asthma on daily life; 3) perceptions of asthma control; 4) exacerbations; and 5) treatment and medication. Questions on symptoms covered the frequency of daytime and nighttime symptoms during the 4 weeks prior to responding the survey, symptoms frequency during the month with the worst asthma control over the past 12 months, and frequency of sudden episodes of severe symptoms. To assess the impact of asthma on daily life, participants were asked about missed work or school days due to asthma, activity limitations, and overall productivity when asthma was at its worst [36–38].

After being carried out in the United States, the AIM was applied to various parts of the world, including LA [35, 37]. In LA, professional interviewers (non-doctors or health professionals) interviewed 51,208 families, from which 2168 individuals with asthma (approximately 400/country) were identified, including those aged 18 or more and parents and/or adults responsible for asthmatic adolescents (ages >12 to 17) from five countries (Argentina, Brazil, Mexico, Puerto Rico, Venezuela) [38]. The household prevalence of asthma among interviewed subjects was: 3.4% in Argentina and in Mexico, 8.8% in Brazil, 16.6% in Venezuela and 18.3% in Puerto Rico. All interviewees answered the standard AIM questionnaire [35–38].

Among analyzed patients, 42% mentioned they had a written asthma control plan, and 50% of these patients had their control plan revised by their doctors at every appointment. Although 34% of the interviewees had heard about peak expiratory flow meters, only 12% used this device [38]. Regarding asthma control medications, 41% of the respondents agreed that they should use them on the long-term and continuously; however, 65% of the interviewees believe that long-term use of medication should only occur when symptoms are present. Sixty percent of the respondents were worried about the daily use of inhaled corticosteroids, and 61% stated that they considered the daily use of rescue inhalers acceptable [38]. The highest proportion of individuals using asthma control medications was found in Puerto Rico (60%), while the lowest proportion was found in Venezuela (35%) [38]. Another very interesting observation was that only 55% of the respondents used inhaled medications. Sixty-one percent of the parents and/or adults responsible for asthmatic children stated they had interrupted children’s asthma control medications over one month before the interview [38]. This may explain why at least 20% of the analyzed individuals presented symptoms every day or night or on most days or nights [38].

Generally, interviewees overestimated their asthma control level. When asked about their or their children’s asthma control during the four weeks before the interview, 60% of the interviewees stated that their asthma was completely-controlled or well-controlled, although only 7% of the respondents reached the criteria established by the GINA for well-controlled asthma (Table 2). Considering symptom frequency, activity limitations and use of asthma relief medication, 35% of the interviewees in the LA AIM may be considered to have uncontrolled asthma [38].

**Table 2:** Distribution of patients with asthma (percentage related to the total of each country) according to their level of asthma control^a^: well-controlled, partly-controlled and uncontrolled in Latin American countries [37, 38]

Country	n	Well-controlled n (%)	Partly-controlled n (%)	Uncontrolled n (%)
Argentina	436	21 (4.8)	211 (48.4)	204 (46.8)
Brazil	399	37 (9.3)	226 (56.6)	136 (34.1)
Mexico	532	50 (9.4)	297 (55.8)	185 (34.8)
Puerto Rico	401	31 (7.7)	235 (58.6)	135 (33.7)
Venezuela	400	12 (3.0)	258 (64.5)	130 (32.5)
Total	2168	151 (7.0)	1227 (56.6)	790 (36.4)

^a^According to Global Initiative for Asthma – GINA [2]

Besides overestimating asthma control levels, interviewees showed low expectations regarding the benefits of successful asthma control. Between 44 and 51% would consider asthma to be well-controlled if they or their children had only two unscheduled medical visits or only one visit to an emergency department [38]. The complementary analysis on the use of the health system by these patients revealed a higher consumption of medications for asthma (OR between 1.6 and 41), a higher number of visits to emergency departments or hospital admissions due to asthma during the previous year (OR between 2.1 and 5.9) and a decrease in productivity among patients with uncontrolled asthma in comparison to patients with well-controlled asthma [39]. This reinforces previous findings of higher costs incurred by these patients to public health systems.

The rate of hospitalizations may serve as an indicator of the illness’ impacts on the population, and, indirectly, as an indicator of the disease level of control. In a survey of annual hospitalizations sensitive to primary care interventions in Brazil between 1998 and 2009, asthma has been found as the third most frequent cause of hospitalizations in the public health system [40]. These ten years have seen a continuous reduction in hospitalization rates, dropping from approximately 23 hospitalizations per 10,000 inhabitants to a little over 10 per 10,000 inhabitants in 2009, with an average annual reduction in hospitalizations of 6.5% among men and 7.7% among women [40]. These good results were certainly influenced by programs providing free asthma medications in Brazil during this period.

## Conclusion

In conclusion, Latin America has a high prevalence and severity of asthma. Although rare, studies on asthma control in Latin America repeatedly show that patients are inadequately controlled and frequently overestimate their degree of asthma control according to the criteria used by international asthma treatment guidelines. It is essential that additional education for doctors and patients be implemented, reinforcing the need for asthma control management focused on the current guidelines, so that more patients may reach adequate control of their illness, thus reducing the individual and social burden of asthma.

## Abbreviations

AIM: Asthma insights and management
AIRLA: Asthma insights and reality in Latin America
EISL: International study of wheezing in infants
EISL-1: EISL phase 1
EISL-3: EISL phase 3
GINA: Global initiative for asthma
ISAAC: International Study of Asthma and Allergy in Childhood
LA: Latin America
OR: Odds ratio
ProAR: Program for control of asthma and allergic rhinitis
SUS: Brazilian system of health
